# *Bombyx* E75 isoforms display stage- and tissue-specific responses to 20-hydroxyecdysone

**DOI:** 10.1038/srep12114

**Published:** 2015-07-13

**Authors:** Kang Li, Enen Guo, Muktadir S. Hossain, Qingrong Li, Yang Cao, Ling Tian, Xiaojuan Deng, Sheng Li

**Affiliations:** 1 Guangdong Provincial Key Laboratory of Agro-animal Genomics and Molecular Breeding/Guangdong Provincial Sericulture and Mulberry Engineering Research Center, College of Animal Sciences, South China Agricultural University, Guangzhou, 510642, China; 2 Key Laboratory of Insect Developmental and Evolutionary Biology, Institute of Plant Physiology and Ecology, Shanghai Institutes for Biological Sciences, Chinese Academy of Sciences, Shanghai 200032, China; 3 The Sericultural and Agri-Food Research Institute of the Guangdong Academy of Agricultural Sciences, Guangzhou 510610, China

## Abstract

Resulted from alternative splicing of the 5′ exons, the nuclear receptor gene *E75* in the silkworm, *Bombyx mori*, processes three mRNA isoforms, *BmE75A*, *BmE75B* and *BmE75C*. From the early 5^th^ larval instar to the prepupal stages, BmE75A mRNA and protein levels in the prothoracic glands display developmental profiles similar to ecdysteroid titer. In the fat body, mRNA levels but not protein levels of all three BmE75 isoforms correlate with ecdysteroid titer; moreover, proteins of all three BmE75 isoforms disappear at the prepupal stages, and a modified BmE75 protein with smaller molecular weight and cytoplasm localization occurs. At the early 5^th^ larval instar stage, treatment of the prothoracic glands and fat body with 20-hydroxyecdysone (20E) and/or cycloheximide (CHX) revealed that BmE75A is 20E primary-responsive at both mRNA and protein levels, while BmE75B and BmE75C exhibit various responses to 20E. At the early wandering stage, RNAi-mediated reduction of gene expression of the 20E nuclear receptor complex, *EcR*-*USP*, significantly decreased mRNA and protein levels of all three BmE75 isoforms in both tissues. In conclusion, BmE75 isoforms display stage- and tissue-specific responses to 20E at both mRNA and protein levels; moreover, they are regulated by other unknown factors at the protein level.

The molting hormone (20-hydroxyecdysone, 20E) determines major developmental transitions in insects, including molting and metamorphosis. 20E binds to a heterodimer of nuclear receptors, the ecdysone receptor (EcR) and Ultraspiracle (USP), to trigger a transcriptional cascade, including several transcription factor genes (i.e, *Br-C*, *E74*, *E75*, *E93*, *HR3* and *βFTZ-F1*) and their downstream genes^1^,^2^.

The structure of a typical nuclear receptor comprises an N-terminal activation domain AF-1, a DNA binding domain (DBD), a hinge region, a conserved ligand binding domain (LBD) and a variable C-terminal activation domain AF-2^3^,^4^. Many small lipophilic molecules such as hormones, fatty acids, bile acids, retinoid acids and vitamins have been identified as ligands of nuclear receptors. The nuclear receptors without identified ligands are often called orphan nuclear receptors^4^,^5^. *E75* was first identified as a 20E primary-response gene in the fruit fly, *Drosophila melanogaster*^6^,^7^. For a long time, E75 was believed to be an orphan nuclear receptor. Until 10 years ago, it was found that the LBD of E75 binds to heme that responds to gas (NO and CO) binding^8^,^9^,^10^. The E75 homologues in vertebrates are Rev-erb α (NR1D1) and Rev-erb β (NR1D2), which are involved in the regulation of circadian rhythm^11^,^12^,^13^.

In *Drosophila*, there are four *DmE75* mRNA isoforms*, DmE75A*, *DmE75B*, *DmE75C* and *DmE75D*, which originate from a single copy gene through differential promoter usage and alternative splicing of 5′ exons. The DBDs of DmE75A and DmE75C contain two C4 zinc fingers, the DBD of DmE75B has one zinc finger, while DmE75D lacks a DBD^7^,^14^,^15^,^17^. 20E rapidly and abundantly induces expression of *DmE75A* and *DmE75B*, whereas its induction of *DmE75C* expression is slow and weak; moreover, juvenile hormone (JH) also induces *DmE75A* expression^16^,^17^. Germ-line clones of *DmE75*-null mutants missing all three isoforms lead to the arrest during mid-oogenesis^16^,^18^. Isoform-specific *DmE75* null mutants exhibit different phenotypes: *DmE75A* mutants show reduced ecdysteroid titer leading to developmental retardation and molting defects, *DmE75B* mutants can survive and exhibit normal reproductive performance, whereas *DmE75C* mutants die within a few days after eclosion^16^. Gain-of-function studies revealed that DmE75B might act as a repressor by reversing the transcriptional activation of its heterodimeric partner DmHR3^19^. However, lose-of-function of *DmE75B* has no effects on DmHR3-induced *DmβFtz-F1* expression and developmental transition^16^. This could be because that DmE75A and DmE75B equally interact with DmHR3 to inhibit *DmβFtz-F1* expression; and the function of DmE75A and DmE75B to inhibit the transcriptional activity of DmHR3 can be reversed by NO^8^,^9^. However, in female adults, DmE75A induces apoptosis in the egg chamber at stages 8 and 9, while DmE75B prevents DmE75A function and thus allows egg development, showing opposite roles in regulating female reproduction^20^. In addition, in the absence of 20E, the heme-binding DmE75A might compete with DmEcR-DmUSP for binding DNA in promoter regions of 20E primary-response genes and thus repress 20E signaling^21^.

In the silkworm, *Bombyx mori*, two isoforms of *BmE75*, *BmE75A* and *BmE75C*, were reported a decade ago. Similar to the findings in *Drosophila*, 20E rapidly and abundantly induces *BmE75A* expression in the ovary, while its induction of *BmE75C* expression is slow and weak. Consequently, *BmE75A* expression proceeds with *BmE75C* expression during ovary development^22^. Moreover, both BmE75A and BmE75C interact with BmHR3 and repress the transcriptional activity of BmHR3 to induce retinoic acid receptor-related receptor response element (RORE) linked target genes expression^23^. Recently, large scale full-length cDNA sequencing reveals the third *BmE75* isoform, *BmE75B*^24^. In this study, we discovered that, at both mRNA and protein levels, BmE75 isoforms display different developmental profiles in response to 20E in the prothoracic glands and fat body. In addition to 20E, other unknown factors are involved in the regulation of BmE75 isoforms at the protein level.

## Results

### Three BmE75 isoforms

**Figure 1:**
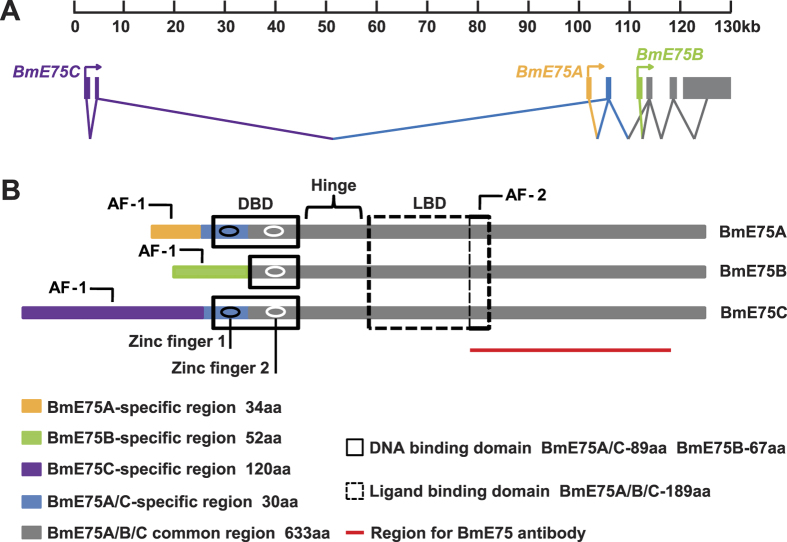
Three BmE75 isoforms. (**A**) Genomic location of the three transcripts of BmE75. Gray boxes denote common 3′ exons. Purple, yellow and green boxes denote specific 5′ exons of *BmE75A*, *BmE75B* and *BmE75C*, respectively. Diagram of BmE75A, BmE75B and BmE75C protein structures. Different colors denote protein domains correspond to the product of exons in (**A**). DBD, DNA binding domon; LBD, ligand binding domon; AF-1, activitiaon function-1; AF-2, activitiaon function-2. The red line shows the region to generate the BmE75 antibody.

As shown in SilkDB (http://silkworm.genomics.org.cn/silkdb/), the *BmE75* gene locus is located on chromosome 10, spanning 130 kb of genomic DNA. Each *BmE75* isoform is characterized by a unique N-terminal sequence encoded by one (*BmE75A* and *BmE75B*) or two (*BmE75C*) distinct 5′ exons. These 5′ exons splice to a common set of four 3′ exons for *BmE75A* and *BmE75C*, while *BmE75B* shares only the last three 3′ exons (Fig. 1A). Overall, the three *BmE75* isoforms, *BmE75A*, *BmE75B* and *BmE75C*, originate from a single copy gene through differential promoter usage and alternative splicing of 5′ exons, showing similar arrangement of gene structure to *DmE75*^7^,^14^,^15^,^16^.

As a result of the arrangement of the *BmE75* gene locus, the predicted molecular weights of BmE75A, BmE75B and BmE75C are 77, 76 and 83 kDa, respectively. Conservative domain analysis reveals that all three BmE75 isoforms contain DBD and LBD that are canonical domains of nuclear receptors. The DBD of BmE75A and BmE75C (89 aa) contains two C4 zinc fingers for binding to DNA in promoter regions of target genes to regulate gene expression, while the DBD of BmE75B (67 aa) contains only one C4 zinc finger and is thus incapable of binding to DNA. The AF-1 of BmE75A, BmE75B and BmE75C varies in sequence and length, implying that they recruit different co-activators or co-repressors onto target gene promoters to regulate gene expression in a ligand-independent manner^25^. All three BmE75 isoforms have the same C-termini, including the hinge region, LBD and AF-2 as well as a portion of the DBD (Fig. 1B). Again, the protein structures of BmE75 isoforms are similar to those of DmE75 isoforms^7^,^14^,^15^,^16^.

### Verification of the BmE75 antibody

**Figure 2:**
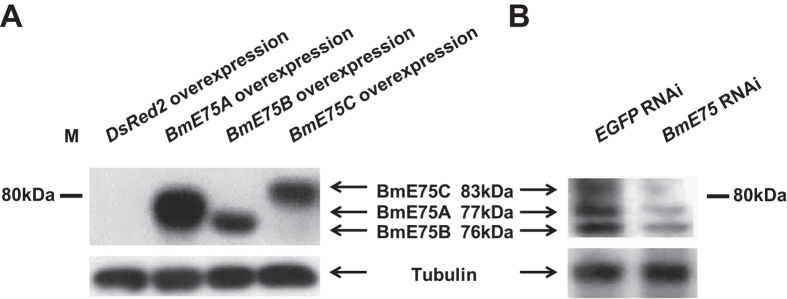
Verification of the BmE75 antibody. (**A**)Baculovirus-mediated overexpression of *BmE75A, BmE75B* and *BmE75C* in Sf9 cells. *DsRed2* overexpression was used as a control. After generation of P2 virus, the Sf9 cells were collected and prepared for Western blotting using the BmE75 antibody. (**A**) dsRNA targeting a common region of all three *BmE75* isoforms was injected into *Bombyx* larvae at the initiation of the wandering stage, the fat body was isolated 24 hours after RNAi and prepared for Western blotting using the BmE75 antibody. *EGFP* dsRNA was used as a control.

To understand BmE75 isoforms at the protein level, an antibody was generated against a portion of their common C-termini (Fig. 1B). Both gain-of-function and lose-of-function studies were employed to verify whether the BmE75 antibody was able to simultaneously detect all three isoforms by Western blotting. The three BmE75 isoforms were individually overexpressed in Sf9 cells using the baculovirus-mediated expression system. Western blotting revealed that the molecular weights of BmE75A, BmE75B and BmE75C are 77, 76 and 83 kDa, respectively, in consistent with their predicted molecular weights (Fig. 2A). Importantly, the overexpressed BmE75A, BmE75B and BmE75C have the same molecular weights as their endogenous proteins isolated from the fat body at the wandering stage (Fig. 2B). Moreover, using dsRNA targeting the common region of all three *BmE75* isoforms at the initiation of the wandering stage, RNAi-mediated reduction of *BmE75* expression lowered the protein levels of all there BmE75 isoforms in the fat body 24 hours after RNAi (Fig. 2B). The above experiments confirmed the authenticity of the BmE75 antibody, which was used throughout the paper.

### BmE75A and BmE75B are 20E primary-responsive in BmN cells

**Figure 3:**
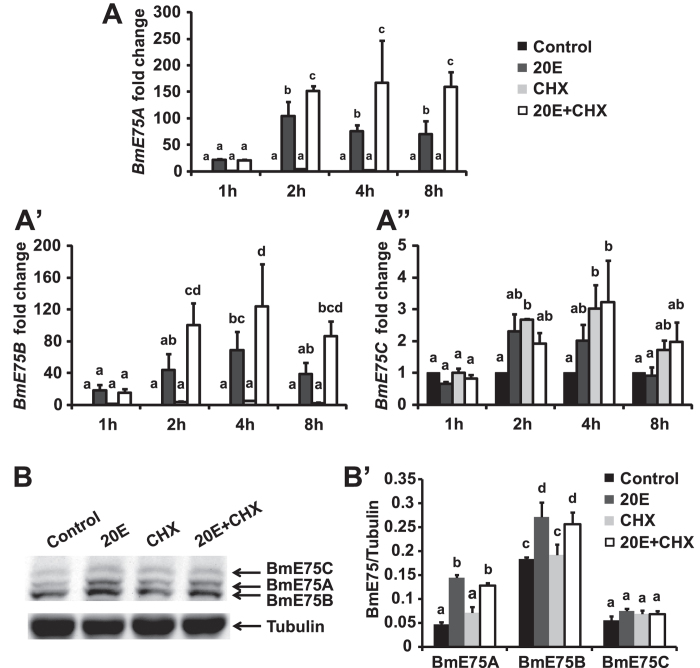
Responses of BmE75 isoforms to 20E in BmN cells. (**A-A”**) Responses of mRNA levels of BmE75A (**A**), BmE75B (**A’**) and BmE75C (**A”**) to 2 μM 20E or/and 10 μg/ml cycloheximide (CHX) in BmN cells. (B-B’) Response of protein levels of BmE75A, BmE75B and BmE75C to 20E or/and CHX in BmN cells 2 hours after treatment. (**B’**) Quantification of BmE75A, BmE75B and BmE75C protein levels in (**B**).

To examine how *BmE75* isoforms respond to 20E, we treated ovary-derived *Bombyx* BmN cells with 20E in the presence or absence of a protein synthesis inhibitor, cycloheximide (CHX). As determined by quantitative real-time PCR (qPCR), mRNA levels of *BmE75A* and *BmE75B* increased approximately 15-fold 1 hour after 20E treatment, no matter whether CHX was present or absent (Figs. 3A, A’). *BmE75A* mRNA level continued increasing to approximately 100-fold 2 hours after 20E treatment and the increase slightly dropped at the next hours (Fig. 3A). Meanwhile, *BmE75B* mRNA level continued increasing to approximately 60-fold 4 hours after 20E treatment and the increase slightly dropped at the next hours (Fig. 3A’). Importantly, CHX did not affect expression of *BmE75A* and *BmE75B*; moreover, the 20E-induced expression of *BmE75A* and *BmE75B* was not blocked by CHX, indicating that both isoforms are 20E primary-responsive in BmN cells (Figs. 3A, A’). By contrast, mRNA level of *BmE75C* did not change 1 hour after 20E treatment, increased only 2-fold 2 and 4 hours after 20E treatment, and decreased to the initial level 8 hours after 20E treatment. Interestingly, despite that CHX also weakly induced *BmE75C* expression, CHX and 20E together did not show overlapping induction of *BmE75C* expression (Fig. 3A”). Overall, 20E rapidly and abundantly induces expression of *BmE75A* and *BmE75B* in BmN cells, while its induction of *BmE75C* expression is slow and weak, showing similar results observed in the *Bombyx* ovary^22^.

We next examined how 20E and CHX affect protein levels of all three BmE75 isoforms in BmN cells. As detected by Western blotting, BmE75A and BmE75B protein levels increased approximately 3- and 1.5-fold 2 hours after 20E treatment with or without CHX, while 20E and/or CHX had no effects on BmE75C protein level (Fig. 3B, B’). These results demonstrated that BmE75A and BmE75B are 20E primary-responsive at both mRNA and protein levels in BmN cells, while BmE75C is not 20E primary-responsive.

### Developmental profiles of BmE75 isoforms in prothoracic gland

**Figure 4:**
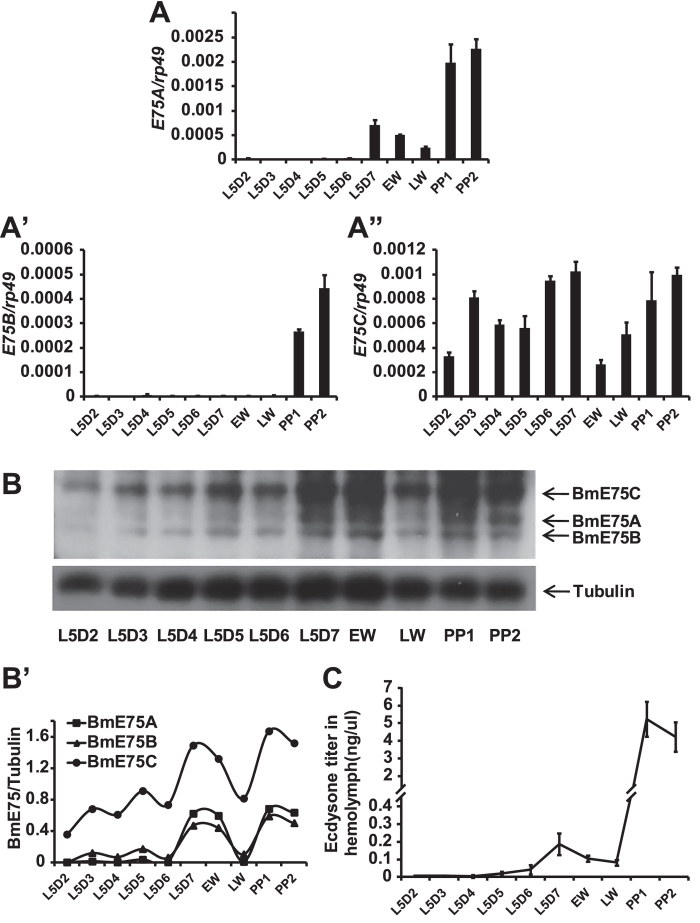
Developmental profiles of BmE75 in prothoracic glands. (**A-A”**) Developmental profiles of mRNA levels of *BmE75A* (**A**), *BmE75B* (**A’**) and *BmE75C* (**A”**) in the prothoracic glands from day 2 of the 5^th^ larval instar (L5D2) to day 2 of the prepupal stage (PP2). L, instar; D, day; EW, early wandering; LW, late wandering; PP, prepupal; P, pupa. (**B-B’**) Developmental profiles of protein levels of BmE75A, BmE75B and BmE75C in the prothoracic glands from L5D2 to PP2. The full-length blots are presented in Supplementary Figure 1. (**B’**) Quantification of BmE75A, BmE75B and BmE75C protein levels in (**B**). Developmental profile of ecdysteroid titer in the hemolymph measured by radioimmunoassay from L5D2 to PP2.

The prothoracic glands produce and secrete ecdysone, the immediate precursor of 20E during insect larval development^26^. From day 2 of the 5^th^ larval instar (L5D2) to day 2 of the prepupal stage (PP2), the developmental expression profiles of all three BmE75 isoforms in the prothoracic glands were examined at both mRNA and protein levels. *BmE75A* mRNA level is under detectable during the feeding larval stages, and reaches a small peak on L5D7 and a very high peak on PP1 and PP2 (Fig. 4A). *BmE75B* mRNA level is under detectable during the feeding larval stages and the wandering stages, and reaches a peak on PP1 and PP2 (Fig. 4A’). Differently, *BmE75C* is continuously expressed from L5D2 and PP2 with relatively high expression on L5D7 and PP2 (Fig. 4A”).

BmE75A and BmE75B protein levels are low, and gradually increase from L5D2 to L5D6, reach the first peak on L5D7 and the early wandering stage (EW) and the second peak on PP1 and PP2. BmE75C protein level is much higher than BmE75A and BmE75B protein levels and can be always detected during the fifth instar, but the developmental profiles of protein levels of all three BmE75 isoforms are quite similar to one another (Fig. 4B, B’). Notably, the mRNA peaks of all the BmE75 isoforms are much more significant than their individual protein peaks.

Since the BmE75 isoforms are responsive to 20E treatment in BmN cells, we questioned whether the developmental profiles of the BmE75 isoforms are consistent with that of ecdysteroid titer. As measured by radioimmunoassay (RIA), a small peak of ecdysteroid titer was detected on L5D7, and a big peak was detected on PP1 (Fig. 4C). In general, BmE75A mRNA and protein levels in the prothoracic glands display developmental profiles similar to ecdysteroid titer, while the developmental profile of BmE75C mRNA level is different from that of ecdysteroid titer.

### Developmental profiles of BmE75 isoforms in fat body

**Figure 5:**
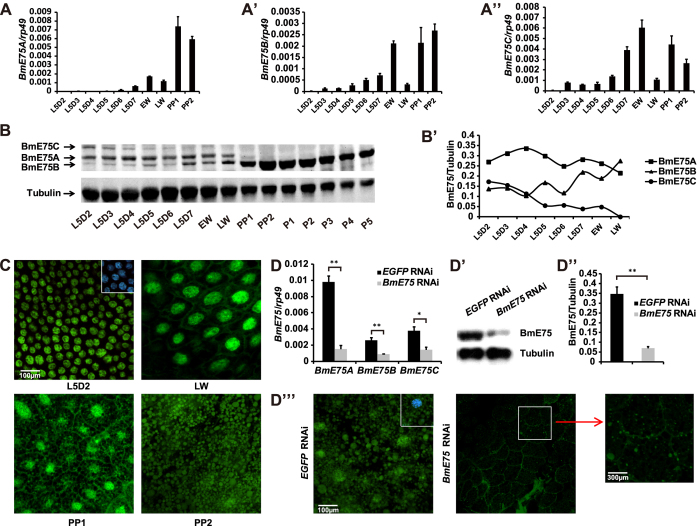
Developmental profiles of BmE75 in fat body. (**A-A”**) Developmental profiles of mRNA levels of *BmE75A* (**A**), *BmE75B* (**A’**) and *BmE75C* (**A”**) in the fat body from day 2 of the 5^th^ larval instar (L5D2) to day 2 of the prepupal stage (PP2). L, instar; D, day; EW, early wandering; LW, late wandering; PP, prepupal; P, pupa. (**B-B’**) Developmental profiles of protein levels of BmE75A, BmE75B and BmE75C in the fat body from L5D2 to P5. The full-length blots are presented in Supplementary Figure 2. (**B’**) Quantification of BmE75A, BmE75B and BmE75C protein levels in (**B**). Location of BmE75 proteins in the fat body is detected by immunohistochemistry using the BmE75 antibody from EW to PP2 stage (green). The white box at top-right corner of inset in (L5D2) is merged with DAPI (blue) staining. (**D-D”’**) dsRNA targeting a common region of all three *BmE75* isoforms was injected into *Bombyx* larvae at EW, and the fat body was isolated 48 hours after treatment. *EGFP* dsRNA was used as a control. mRNA levels of *BmE75A*, *BmE75B* and *BmE75C* were detected using isoform-specific primers by qPCR (**D**). Western blotting (**D’,D”**) and immunohistochemistry (**D”’**) were performed to detect the small molecular weight BmE75-like protein in the fat body using the BmE75 antibody (green). The white box at top-right corner of inset in (*EGFP* RNAi) in (**D”’**) is merged with DAPI (blue) staining.

The fat body is an organ analogue to vertebrate adipose tissue and liver and functions as a major organ for nutrient storage and energy metabolism in insects^27^. *BmE75A* mRNA level in the fat body is under detectable during the feeding larval stages, and reaches a small peak on L5D7 and a very high peak on PP1 and PP2 (Fig. 5A). *BmE75B* and *BmE75C* mRNA levels show similar developmental profiles to *BmE75A* mRNA level, while they gradually increase from L5D2 to L5D6 (Fig. 5A’). The results show that, in the fat body, mRNA levels of all three BmE75 isoforms correlate with ecdysteroid titer.

Unexpectedly, it appears that protein levels of all three BmE75 isoforms in the fat body show no correlations with ecdysteroid titer. All of them are detectable from L5D2 to the late wandering stage (LW), with no apparent change for BmE75A protein level, a slight increase for BmE75B protein level, and a steady decrease for BmE75C protein level (Fig. 5B, B’). To our surprise, proteins of all three BmE75 isoforms disappear at the prepupal stages, and a BmE75-like protein with smaller molecular weight occurs at the prepupal stages and persists throughout the pupal stages (Fig. 5B). As monitored by immunohistochemistry, BmE75 proteins are located at the nuclei of fat body cells from L5D2 to LW, at both nuclei and cytoplasm at PP1, and only at cytoplasm at PP2 (Fig. 5C). The immunohistochemistry studies show that the BmE75-like protein is located at cytoplasm rather than nuclei.

To confirm whether the small molecular weight protein band is relevant to BmE75, using dsRNA targeting the common region of all three *BmE75* isoforms, RNAi-mediated reduction of *BmE75* expression was performed at the initiation of the wandering stage. Forty-eight hours after RNAi, mRNA levels of all three *BmE75* isoforms (Fig. 5D), the small molecular weight protein band (Fig. 5D’), and immunostaining in both nuclei and cytoplasm (Fig. 5D”’) significantly decreased, indicating that it is a modified BmE75 protein with smaller molecular weight.

### BmE75 isoforms display tissue-specific responses to 20E

Since significant differences were observed between the prothoracic glands and fat body with respect to the developmental profiles of BmE75 isoforms at both mRNA and protein levels, we examined whether BmE75 isoforms display tissue-specific responses to 20E. The prothoracic glands and fat body were isolated form L5D2 larvae, cultured *in vitro*, treated with 20E in the presence or absence of CHX, and mRNA and protein levels of BmE75 isoforms were measured 2 hours after treatment.

**Figure 6:**
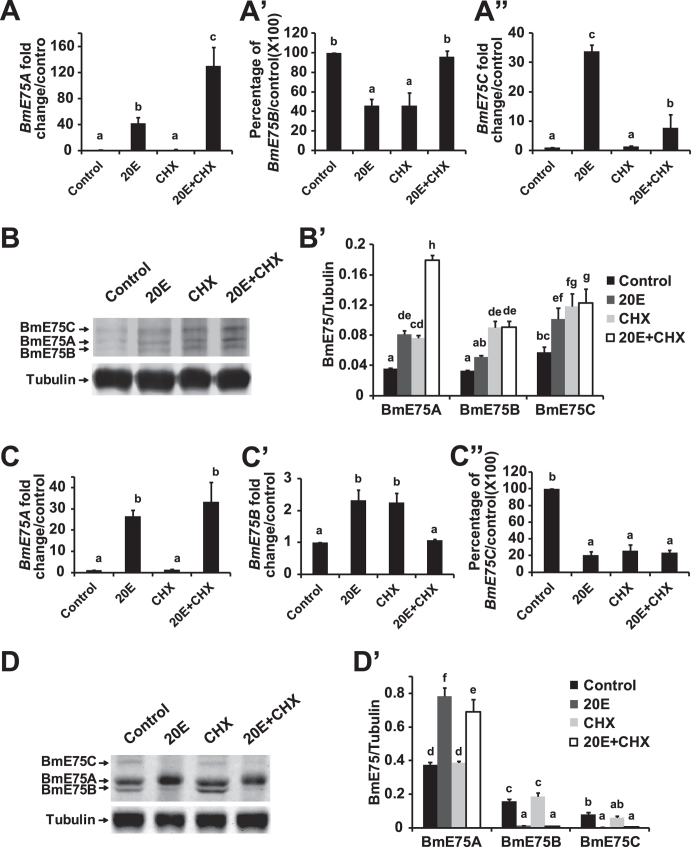
Tissue-specific responses of BmE75 isoforms to 20E on L5D2. (**A-A”**) Responses of mRNA levels of *BmE75A* (**A**), *BmE75B* (**A’**) and *BmE75C* (**A”**) to 2 μM 20E or/and 10 μg/ml cycloheximide (CHX) in the prothoracic glands on day 2 of the fifth instar (L5D2). The mRNA levels were measured 2 hours after treatment *in vitro*. (**B-B’**) Response of protein levels of BmE75A, BmE75B and BmE75C to 20E or/and CHX in the L5D2 prothoracic glands 2 hours after treatment. (**B’**) Quantification of BmE75A, BmE75B and BmE75C protein levels in (**B**). (**C-D’**) The same as (**A-B’**) except the fat body was used.

In the prothoracic glands, *BmE75A* mRNA levels increased approximately 40-fold by 20E, CHX did not affect *BmE75A* expression, and the 20E induction was not blocked by CHX, indicating that *BmE75A* is 20E primary-responsive in this tissue (Fig. 6A). 20E or CHX decreased half of *BmE75B* mRNA levels, while 20E and CHX together showed no effects on *BmE75B* expression (Fig. 6A’). Despite that *BmE75C* mRNA levels increased approximately 30-fold by 20E, the 20E induction was significantly blocked by CHX, indicating that *BmE75C* is 20E secondary-responsive in this tissue (Fig. 6A”). Meanwhile, protein levels of BmE75A, BmE75B and BmE75C increased approximately 2.2-, 1.4- and 1.7-fold by 20E, and the 20E induction was not blocked by CHX (Fig. 6B, B”).

In the fat body, *BmE75A* is 20E primary-responsive as well (Fig. 6C). 20E or CHX increased *BmE75B* mRNA levels by 2-fold, while 20E and CHX together had no effects on *BmE75B* expression (Fig. 6C’). 20E and/or CHX decreased *BmE75C* mRNA levels to approximately 20% of the control levels (Fig. 6C”). Meanwhile, protein levels of BmE75A increased approximately 2-fold by 20E, and the 20E induction was not blocked by CHX. Meanwhile, protein levels of BmE75B and BmE75C decreased to very low levels by 20E, and CHX had no effects (Fig. 6D, D’).

In conclusion, BmE75A is 20E primary-responsive in the prothoracic glands and fat body at both mRNA and protein levels, while BmE75B and Bm75C exhibit various responses to 20E. The experimental data in BmN cells, the prothoracic glands and fat body also show that mRNA levels of all three BmE75 isoforms respond to 20E more dramatically than their protein levels, indicating that BmE75 isoforms are regulated by other unknown factors at the protein level.

### Attenuation of 20E signaling decreases all BmE75 isoforms in both tissues

**Figure 7:**
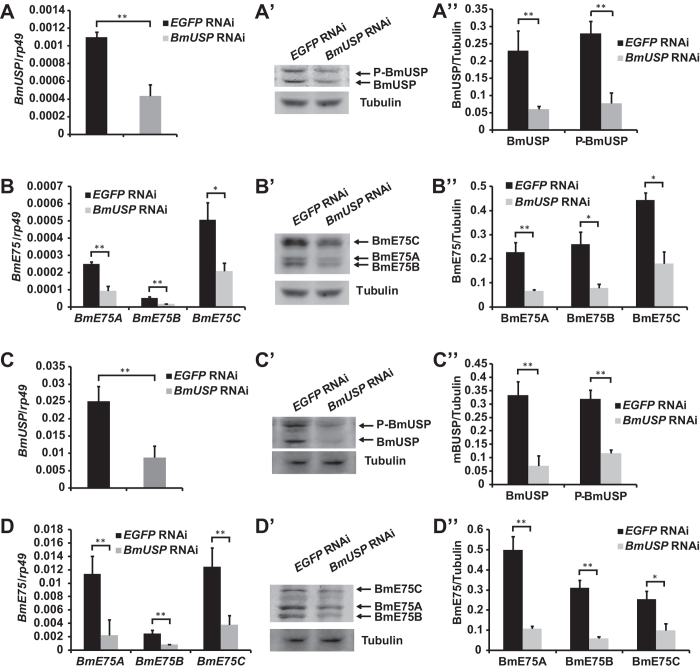
Attenuation of 20E signaling decreases all BmE75 isoforms in both prothoracic glands and fat body during larval-pupal metamorphosis. (**A-B”**) dsRNA of *BmUSP* was injected into *Bombyx* lava at the initiation of the wandering stage (EW), and the prothoracic gland was isolated 24 hours after RNAi. The mRNA and protein levels of BmUSP/phosphorylated BmUSP (P- BmUSP) were detected by qPCR (**A**) and Western blotting (**A’, A”**), and the mRNA and protein levels of BmE75A, BmE75B and BmE75 C were also detected by qPCR (**B**) and Western blotting (**B’, B”**). (**C-D”**) The same as (**A-B”**) except the fat body was used.

Finally, we examined whether 20E signaling is absolutely required for expression of BmE75 isoforms at both mRNA and protein levels during larval-pupal metamorphosis in *Bombyx*. In a previous study, we documented that RNAi knockdown of BmEcR-BmUSP at the initiation of the wandering stage resulted in significant prepupal or pupal lethality and a decrease of 20E signaling, with *BmUSP* RNAi exhibiting stronger inhibitory effects than *BmEcR* RNAi^28^,^29^. RNAi-mediated reduction of *BmUSP* expression was performed at the initiation of the wandering stage, and the effects were determined 24 hours after RNAi. In the prothoracic glands, *BmUSP* RNAi not only decreased BmUSP mRNA (Fig. 7A) and protein (Fig. 7A’) levels, but also decreased mRNA (Fig. 7B) and protein (Fig. 7B’) levels of all three BmE75 isoforms. Similar results were also observed in the fat body (Fig. 7C-7D”). The *BmUSP* RNAi experimental data demonstrated that attenuation of 20E signaling decreases mRNA and protein levels of all BmE75 isoforms in prothoracic glands and fat body during larval-pupal metamorphosis.

## Discussion

### Regulation of E75 isoforms at mRNA level: 20E and beyond

In *Drosophila*, mRNAs of *DmE75* isoforms are responsive to 20E, although differences exist among different isoforms with respect to the kinetics of induction and the sensitivity of the promoters to 20E^14^,^15^. Similar observations were reported for the tobacco hornworm, *Manduca sexta*^30^, the yellow fever mosquito, *Aedes aegypti*^31^,^32^, and the German cockroach, *Blattella germanica*^33^.

In BmN cells (Fig. 3A), the prothoracic glands (Fig. 6A), fat body (Fig. 6C) and ovary^22^, *BmE75A* mRNA is always 20E primary-responsive. Accordingly, the developmental profiles of *BmE75A* mRNA in both prothoracic glands (Fig. 4A) and fat body (Fig. 5A) correlate well with ecdysteroid titer (Fig. 4C). Differently, mRNA levels of *BmE75B* and *BmE75C* display different responses to 20E in stage- and tissue-specific manners. *BmE75B* mRNA is 20E primary-responsive in BmN cells (Fig. 3B), suppressed by 20E in the prothoracic glands on L5D2 (Fig. 6A’), and weakly induced by 20E in the fat body on L5D2 (Fig. 6C’). The developmental profiles of *BmE75B* mRNA in both prothoracic glands (Fig. 4A’) and fat body (Fig. 5A’) correlate with ecdysteroid titer, although no expression was detected in the prothoracic glands on L5D7 (Fig. 4A’). Meanwhile, *BmE75C* mRNA is weakly induced by 20E in BmN cells (Fig. 3A”), 20E primary-responsive in the prothoracic glands on L5D2 (Fig. 6A”), suppressed by 20E in the fat body on L5D2 (Fig. 6C”). The developmental profiles of *BmE75C* mRNA correlate with ecdysteroid titer in the fat body (Fig. 5A”) but not in the prothoracic glands (Fig. 4A”). In spite of all the differences, attenuation of 20E signaling decreases mRNA levels of all BmE75 isoforms in both tissues during larval-pupal metamorphosis (Fig. 7). Since the E75 isoforms have different promoter regions (Fig. 1), it is reasonable that they differently respond to 20E at the mRNA level. Remarkably, BmE75 isoforms display stage- and tissue-specific responses to 20E at the mRNA level. It is likely that a variety of transcriptional cofactors are involved in the fine regulation of *BmE75* mRNA expression in a variety of stages and tissues, depending on the presence or absence of 20E.

### The protein of BmE75 isoforms are regulated at multiple levels

Taken the advantage of BmE75 antibody (Fig. 2), we were able to investigate how BmE75 is regulated by 20E and other factors at the protein level. In BmN cells (Fig. 3B, B’), the prothoracic glands (Fig. 6B, B’) and fat body (Fig. 6D, D’), BmE75A protein is always the same 20E primary-responsive as *BmE75A* mRNA. However, *BmE75A* mRNA level responds to 20E much more dramatically than its protein level, indicating that other unknown factors are involved in the regulation of BmE75A protein level. Although both BmE75B mRNA and protein levels are induced by 20E in BmN cells (Fig. 3), the effects on BmE75B mRNA and protein levels are opposite in the prothoracic glands (Fig. 6A-6B’) and fat body (Fig. 6C-6D’). Meanwhile, 20E slowly and weakly induced *BmE75C* mRNA expression but had no effects on BmE75C protein level in BmN cells (Fig. 3); both BmE75C mRNA and protein levels are induced by 20E in the prothoracic glands (Fig. 6A-6B’) and inhibited by 20E in the fat body (Fig. 6C-6D’). Therefore, mRNA and protein levels of BmE75B and Bm75C show various responses to 20E. Importantly, attenuation of 20E signaling decreases both mRNA and protein levels of all BmE75 isoforms in prothoracic glands and fat body during larval-pupal metamorphosis (Fig. 7), indicating that 20E signaling is indispensable for BmE75 expression at this stage at least. It is necessary to note that the 20E treatments might disturb the naturally physiological conditions in both tissues on L5D2 (no 20E at this stage). In future studies, we should not neglect the stage- and tissue-specific responses to 20E when designing, performing and explaining gain-of-function studies.

In consistent with their mRNA levels, the developmental profiles of protein levels of all three BmE75 isoforms in the prothoracic glands (Fig. 4) correlate with ecdysteroid titer. By contrast, despite mRNA levels of all three BmE75 isoforms correlate with ecdysteroid titer in the fat body, it appears that their protein levels show no correlations with ecdysteroid titer. Instead, a modified BmE75 protein with smaller molecular weight occurs at the prepupal stages and persists throughout the pupal stages (Fig. 5). Because the proteins of all three BmE75 isoforms are modified, it is impossible to judge whether they have actual correlation with ecdysteroid titer in the fat body during the prepual and pupal stages.

Besides the regulation of BmE75 mRNA levels by 20E, what other factors might regulate BmE75 protein levels? Presumably, any posttranscriptional modification mechanisms might affect BmE75 protein levels. For example, microRNA might affect translation efficiency^34^,^35^; we found that the common 3′ end of all three *BmE75* isoforms contain multiple possible target sites of several microRNAs. It was reported that nuclear receptors undergo posttranslational modifications, including phosphorylation, sumoylation, acetylation and ubiquitination^36^,^37^. In our laboratory, we have discovered that DmUSP is modified by phophorylation and sumoylation, which play roles to maintain its protein stability^38^,^39^. For example, a putative casein kinase II phosphorylation site and multiple sumoylation sites are predicted in all three BmE75 isoforms, but we currently do not know whether these sites have effects on BmE75 protein levels. Presumably, these potential regulatory factors affecting BmE75 protein levels are stage- and tissue-specific as well. To identify those factors should be of great importance to understand the regulation of BmE75 protein levels and probably 20E signaling in general.

### A modified BmE75 protein with smaller molecular weight in the fat body

Probably the most important discovery in this study is the modified BmE75 protein with smaller molecular weight and cytoplasm localization in fat body cells, which occurs at the prepupal stages and persists throughout the pupal stages (Fig. 5). Since proteins of all three BmE75 isoforms disappear at the prepupal and pupal stages, it is likely the N-termini of all three BmE75 protein isoforms are truncated at the same site via a specific proteinase. This truncation should happen at their common DBD or hinge region (Fig. 1A), since a nuclear export signal is predicted at the end of the LBD of BmE75. During larval-pupal metamorphosis, the *Bombyx* fat body undergoes massive PCD including autophagy and apoptosis to eliminate larval tissues and superfluous proteins^40^,^41^, which is mainly regulated by 20E. It will be of interest to investigate how the modified BmE75 protein is formed and what role it might play in the 20E-induced PCD.

Previous studies in *Drosophila* have demonstrated that DmE75 is involved in the regulation of ecdysteroid titer^16^ and female reproduction^20^ by interacting with DmHR3^8^,^9^,^16^,^19^ or DmEcR-DmUSP^21^. We are currently examining the physiological functions and molecular mechanisms of all three BmE75 isoforms in the regulation of *Bombyx* development, with a particular focus on the modified BmE75 protein.

## Materials and Methods

### Silkworm

*Bombyx* larvae (p50 strain) provided by the Sericultural Research Institute, Chinese Academy of Agricultural Sciences (Zhenjiang, China), were reared with fresh mulberry leaves at 25 °C under 14 h light/10 h dark cycles^41^.

### Collection of prothoracic glands, fat body and hemolymph samples

The prothoracic glands, peripheral fat body tissues from the 5^th^ abdominal segment, and hemolymph samples were collected at various developmental stages or after various treatments.

### qPCR

Total RNA was isolated using TRIzol (Invitrogen) according to the manufacturer’s instructions. qPCR was performed as previously described^28^. *rp49* is used as a reference gene that has been validated for qPCR analysis in *Bombyx*^42^.

### Western blotting

A cDNA fragment shared by all three *BmE75* isoforms (Fig. 1B) was expressed in *E. coli* and the purified protein was used for generating a rabbit polyclonal antibody by the Abmart Company (Shanghai, China). AB11 USP antibody was a kind gift from Dr. Fotis Kafatos. The primary antibodies, BmE75 (1:5000), AB11 USP (1:2000) and tubulin (#AT819, Beyotime, China; 1:10000), were used. The Western blotting images were caught by Tanon-5500 Chemiluminescent Imaging System (Tanon, China) / KODAK Medical X-Ray Processor 102 NY14608 (Germany), and quantitative measurements of Western blots were performed using the ImageJ software.

### Radioimmunoassay for measuring ecdysteroid titers

Total ecdysteroid titers of the hemolymph samples were determined by radioimmunoassay (RIA) as described previously^43^. Briefly, hemolymph from different stage *Bombyx* larval were collected, and extracted for ecdysteroids with equal volume of methanol for 3 times. The supernatant after centrifugation was collected and evaporated under 70 °C. RIA was performed to evaluate the supernatants using 20E (Sigma and Aldrich, USA) as a standard. The rabbit antiserum used was raised against 20E conjugated with human serum albumin. [^3^H]-ecdysone (approximately 60 Ci/mmol) was obtained from New England Nuclear (USA). Cross-reactions of the antiserum between ecdysone and 20E occurred at a ratio of 1:2.5.

### Fluorescence microscopy and immunohistochemistry

The fat body tissues were isolated at the indicated times as described previously^44^. For detecting BmE75 proteins by immunohistochemistry, the fat body tissues were fixed in 4% paraformaldehyde for 45 min at room temperature, blocked in phosphate buffered saline containing 5% BSA and 1% Triton-X (PBSBT) for 1 h, and incubated with the anti-BmE75 antibody at a dilution of 1:500 at 4 °C overnight. Fat body tissues were washed for 1 h in PBSBT and incubated with a FITC-(green) conjugated secondary antibody (diluted 1:200) for 2 h. DAPI (Sigma) was added to label nuclei and the stained fat bodies were imaged using an Olympus FV10-ASW confocal microscope at 40× magnification^45^.

### Cell culture and tissue culture

BmN cells were maintained in TC-100 medium (BIOTECH, Germany) supplemented with 10% heat-inactivated fetal bovine serum (FBS) (Gibco). Sf9 cell line was cultured in HyClone SFX medium (Thermo Scientific) supplemented with 5% FBS. Cells were pre-incubated for 1 day before further experiments. For *ex vivo* experiments, newly collected prothoracic gland and fat body tissues from L5D2 larvae were cultured in Grace’s medium (Sigma-Aldrich, 11300-043) at 25 °C. After pre-incubation for 1 h, the medium was replaced with fresh medium. To determine which *BmE75* genes are 20E-primary response genes, 2 μM 20E or/and 10 μg/ml CHX (Enzo Life Science, ALX-380-269) were added. After incubation for the indicated times, mRNA and protein were isolated for qPCR analysis and Western blotting.

### RNA interference of *BmE75* and *BmUSP* in *Bombyx* larvae

For RNAi of *BmE75*, a 644 bp common region was amplified by PCR from cDNA using a pair of primers (forward primer, 5′- GGCTTCTTCCGGCGATCTAT -3′; reverse primer, 5′- CCGCTCTGGATGGAGTCTCTC -3′) with T7 RNA polymerase-binding site (5′-GAATTAATACGACTCACTATAGGGAGA-3′) attached to the 5′-end of each primer. The *BmE75* double-stranded RNA (dsRNA) was synthesized using T7 RiboMAX^TM^ Express RNAi kit (Promega, P1700) according to the manufacturer’s instructions. RNAi of *BmUSP* was conducted as described before^28^. The *EGFP* dsRNA was used as control. Thirty μg dsRNA per larva was injected at the initiation of wandering stage^41^. Prothoracic gland and fat body tissues were collected at the indicated times for further analysis.

### Baculovirus-mediated overexpression of BmE75 isoforms in Sf9 cells

The full-length cDNAs of *BmE75A, BmE75B* and *BmE75C* were cloned into the *Bam*H I - *Hin*d III sites of pFastBac-HTa (Invitrogen) plasmid, and *DsRed2* was used as control^46^. Plasmids were then transformed into DH10Bac^ΔEGT^ bacteria^47^ to prepare bacmid DNA according to the Bac-to-Bac system protocol. Sf9 cells were transfected with bacmid DNA (1 μg/ml) using Cellfectin II transfection reagent (Invitrogen). After 7 days, P1 virus was collected which was then used to prepare P2 virus after another 3 days. Cells infected with the P2 virus were collected and prepared for Western blotting with anti-BmE75 antibody.

### Statistics

The experimental data were analyzed using Student’s *t*-test and ANOVA. For the *t*-test: *p < 0.05; **p < 0.01. ANOVA: bars labeled with different lowercase letters are significantly different (p < 0.05). Throughout the study, values are represented as the mean ± standard deviation of 3–8 independent experiments.

## Additional Information

**How to cite this article**: Li, K. *et al.* *Bombyx* E75 isoforms display stage- and tissue-specific responses to 20-hydroxyecdysone. *Sci. Rep.* **5**, 12114; doi: 10.1038/srep12114 (2015).

## Supplementary Material

Supplementary Information
