# Supplementary material for: Bombyx E75 isoforms display stage- and tissue-specific responses to 20-hydroxyecdysone
Source: Sci Rep. 2015 Jul 13;5:12114. doi: 10.1038/srep12114 (PMC4499807; doi:10.1038/srep12114)
Supplement: Supplementary Information [file srep12114-s1.doc]

***Bombyx* E75 isoforms display stage- and tissue-specific responses to 20-hydroxyecdysone**

**Kang Li^1, 2^, Enen Guo^1, 2^,** **Muktadir** **S. Hossain^2^, Qingrong Li^3^, Yang Cao^1^, Ling Tian^2,*^, Xiaojuan Deng^1,*^, and Sheng Li^2,*^**

^1^ Laboratory of Insect Molecular Biology and Biotechnology, Guangdong Provincial Key Laboratory of Agro-animal Genomics and Molecular Breeding, College of Animal Sciences, South China Agricultural University, Guangzhou 510642, China

^2^ Key Laboratory of Insect Developmental and Evolutionary Biology, Institute of Plant Physiology and Ecology, Shanghai Institutes for Biological Sciences, Chinese Academy of Sciences, Shanghai 200032, China

^3^ The Sericultural and Agri-Food Research Institute of the Guangdong Academy of Agricultural Sciences, Guangzhou 510610, China

Running title: 20E regulation of BmE75 isoforms

^*^ To whom correspondences should be addressed. E-mails: [lisheng01@sibs.ac.cn](mailto:lisheng01@sibs.ac.cn) (SL),

[dengxj@scau.edu.cn](mailto:dengxj@scau.edu.cn) (XD), and [tianling@sibs.ac.cn](mailto:tianling@sibs.ac.cn) (LT); Tel/fax: 86-21-54924163

**Supplementary Figures**


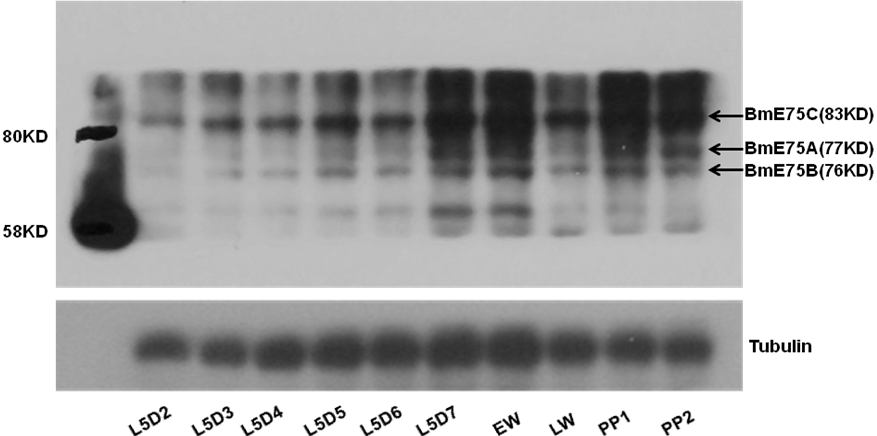


**Figure S1. Developmental profiles of BmE75 in prothoracic glands.**

Developmental profiles of protein levels of BmE75A, BmE75B and BmE75C in the prothoracic glands from L5D2 to PP2. The Western blotting images were caught by KODAK Medical X-Ray Processor 102 NY14608 (Germany).


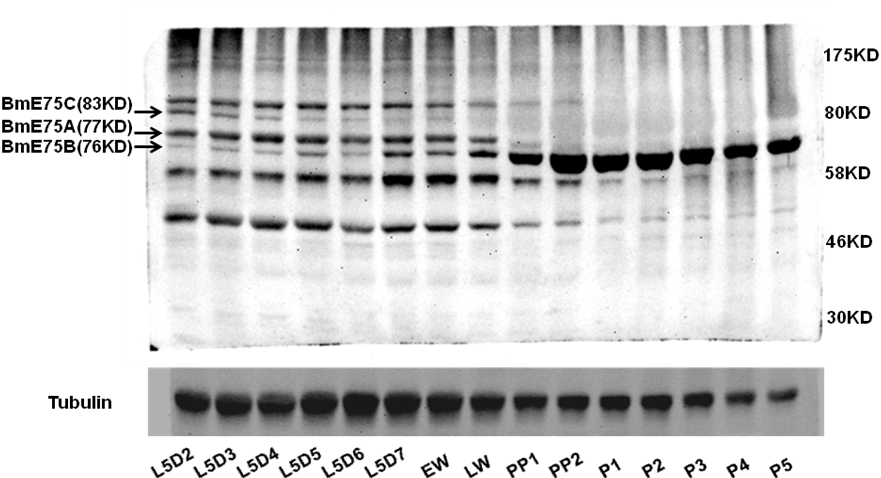


**Figure S2. Developmental profiles of BmE75 in fat body.**

Developmental profiles of protein levels of BmE75A, BmE75B and BmE75C in the fat body from L5D2 to P5. The Western blotting images were caught by Tanon-5500 Chemiluminescent Imaging System (Tanon, China).
